# Supplementary material for: A fault-intrusion-tolerant system and deadline-aware algorithm for scheduling scientific workflow in the cloud
Source: PeerJ Comput Sci. 2021 Nov 2;7:e747. doi: 10.7717/peerj-cs.747 (PMC8576554; doi:10.7717/peerj-cs.747)
Supplement: Supplemental Information 6 [file peerj-cs-07-747-s006.zip › Myproject1.1/docs/org/workflowsim/clustering/balancing/metrics/package-summary.html]

org.workflowsim.clustering.balancing.metrics


JavaScript is disabled on your browser.


- Overview
- Package
- Class
- Use
- Tree
- Deprecated
- Index
- Help

- Prev Package
- Next Package

- Frames
- No Frames

- All Classes

# Package org.workflowsim.clustering.balancing.metrics

- Interface Summary

  | Interface | Description |
  | --- | --- |
  |  |  |
  | --- | --- |
  | BalancingMetric | Every balancing metric should implement this interface |
- Class Summary

  | Class | Description |
  | --- | --- |
  |  |  |
  | --- | --- |
  | DistanceVariance | A DistanceVariance |
  | HorizontalRuntimeVariance | HorizontalRuntimeVariance is the standard deviation of the runtime |
  | ImpactFactorVariance | ImpactFactorVariance is the standard deviation of their impact factors |
  | PipelineRuntimeVariance | The PipelineRuntimeVariance is the standard deviation of the runtime of the pipelines |

- Overview
- Package
- Class
- Use
- Tree
- Deprecated
- Index
- Help

- Prev Package
- Next Package

- Frames
- No Frames

- All Classes
